# Tipranavir/Ritonavir (500/200 mg and 500/100 mg) Was Virologically Non-Inferior to Lopinavir/Ritonavir (400/100 mg) at Week 48 in Treatment-Naïve HIV-1-Infected Patients: A Randomized, Multinational, Multicenter Trial

**DOI:** 10.1371/journal.pone.0144917

**Published:** 2016-01-05

**Authors:** David A. Cooper, Damien V. Cordery, Roberto Zajdenverg, Kiat Ruxrungtham, Keikawus Arastéh, Frank Bergmann, José L. de Andrade Neto, Joseph Scherer, Ricardo L. Chaves, Patrick Robinson, study team

**Affiliations:** 1 The Kirby Institute, University of New South Wales, Sydney, Australia; 2 Head of Medical Affairs, HIV, Infectious Diseases and Immuneinflammatory Diseases, GlaxoSmithKline, Rio de Janeiro, Brazil; 3 HIV-NAT, Thai Red Cross AIDS Research Centre; and Faculty of Medicine, Chulalongkorn University, Bangkok, Thailand; 4 Epimed GmbH, Vivantes Auguste-Viktoria Hospital, Berlin, Germany; 5 Department of Internal Medicine, Infectiology and Pulmonology, Humboldt University, Berlin, Germany; 6 Instituto A Z de Pesquisa E Ensino, Pesquisa E Ensino, Brazil; 7 Boehringer Ingelheim Pharmaceuticals, Inc, Ridgefield, CT, United States of America; 8 Boehringer Ingelheim GmbH, Ingelheim, Germany; Rush University, UNITED STATES

## Abstract

Ritonavir-boosted tipranavir (TPV/r) was evaluated as initial therapy in treatment-naïve HIV-1-infected patients because of its potency, unique resistance profile, and high genetic barrier. Trial 1182.33, an open-label, randomized trial, compared two TPV/r dose combinations versus ritonavir-boosted lopinavir (LPV/r). Eligible adults, who had no prior antiretroviral therapy were randomized to twice daily (BID) 500/100 mg TPV/r, 500/200 mg TPV/r, or 400/100 mg LPV/r. Each treatment group also received Tenofovir 300 mg + Lamivudine 300 mg QD. The primary endpoint was a confirmed viral load (VL) <50 copies/mL at week 48 without prior antiretroviral regimen changes. Primary analyses examined CD4-adjusted response rates for non-inferiority, using a 15% non-inferiority margin. At week 48, VL<50 copies/mL was 68.4%, 69.9%, and 72.4% in TPV/r100, TPV/r200, and LPV/r groups, respectively, and TPV/r groups showed non-inferiority to LPV/r. Discontinuation due to adverse events was higher in TPV/r100 (10.3%) and TPV/r200 (15.3%) recipients versus LPV/r (3.2%) recipients. The frequency of grade ≥3 transaminase elevations was higher in the TPV/r200 group than the other groups, leading to closure of this group. However, upon continued treatment or following re-introduction after treatment interruption, transaminase elevations returned to grade ≤2 in >65% of patients receiving either TPV/r200 or TPV/r100. The trial was subsequently discontinued; primary objectives were achieved and continuing TPV/r100 was less tolerable than standard of care for initial highly active antiretroviral therapy. All treatment groups had similar 48-week treatment responses. TPV/r100 and TPV/r200 regimens resulted in sustained treatment responses, which were non-inferior to LPV/r at 48 weeks. When compared with the LPV/r regimen and examined in the light of more current regimens, these TPV/r regimens do not appear to be the best options for treatment-naïve patients based on their safety profiles.

## Introduction

In the mid 2000s, there were few options available for patients infected with HIV multi resistant to protease inhibitor (PI) antiretroviral drugs (ARVs). During the course of tipranavir’s (TPV) development for treatment of patients with resistant HIV, substantial efficacy was demonstrated [1, 2]. Based on therapies available at the time, approximately 50% of patients receiving first-line therapy would fail by 1 year, chiefly because of emerging resistance [3]. It was hypothesized that first-line treatment with TPV could achieve useful efficacy and might prevent the emergence of PI resistance, because of a high genetic barrier to the emergence of resistance. Therefore, this trial was undertaken in 2004–2006 with the aspiration of avoiding emergence of ARV resistance during first-line therapy.

Combination antiretroviral therapy (cART) has significantly advanced HIV-1 treatment over the past decade, and first-line therapy regimens have become much more effective in achieving and maintaining long-term virologic suppression of plasma HIV RNA [4–10]. However, emergence of resistance remains a concern [4, 11, 12]. Although suppression of HIV replication with first-line therapy is reported in up to 90% cases, virologic treatment failure may still occur in patients treated with cART [4–10, 13–15], limiting treatment options and increasing the risk of death [16]. A substantial prevalence of transmitted ARV resistance among newly infected individuals is well documented [17, 18]; up to 20% of untreated patients may demonstrate resistance to ARV drugs in at least one class [11, 12, 19–22]. Since treatment failure is frequently attributed to the presence or emergence of drug-resistant HIV-1 variants, [23–27] ARVs with unique resistance profiles and high genetic barriers to resistance may improve treatment outcome, by leveraging drugs with unique resistance profiles and higher genetic barriers to resistance.

Based on *in vitro* data demonstrating slow evolution of PI resistance in HIV-1 isolates exposed to TPV [28], this trial was conducted to observe if resistance could be minimized. TPV, co-administered with ritonavir (TPV/r) to attain therapeutic concentrations [29], achieved good virologic responses in a small treatment-naïve study [30] and in two large triple-class-experienced patient studies [1, 2, 31]. TPV/r was safe and well tolerated in the 14-day naïve patient trial [30]. Forty-eight-week results from RESIST-1 and RESIST-2 show that TPV/r 500/200 mg twice daily (BID), along with an optimized backbone regimen (OBR), had superior efficacy in viral suppression and immunologic responses versus an investigator-selected, ritonavir (RTV)-boosted comparator protease inhibitor (CPI/r) in more than 1400 highly treatment-experienced patients [2].

The 1182.33 trial was conducted to evaluate the potential role of TPV/r in treatment-naïve HIV-1-infected adults. In this 48-week, randomized, open-label trial, the efficacy and safety of two dose combinations of TPV/r (500/200 mg or 500/100 mg BID) was compared with the active control group, lopinavir plus ritonavir (LPV/r). Each treatment group also received a standardized nucleoside/nucleotide backbone regimen. In vitro inhibition of wild-type HIV-1 is achieved with lower TPV concentrations than those seen with the 500/200 mg BID dose. Therefore, the lower RTV dose was included for testing because it results in lower TPV concentrations; these lower exposures with a 500/100 mg BID dose were projected to decrease adverse effects of TPV and yet provide adequate inhibitory concentrations. In this report, we provide the final trial data, and put the results into historical and clinical context.

## Materials and Methods

### Patients

HIV-1-infected men and women ≥18 years of age who have never previously been treated with ART (up to 7 days of previous treatment was allowed), CD4+ cell count <500 cells/mm^3^, viral load (VL) ≥5000 copies/mL of plasma HIV-1 RNA at screening, and acceptable laboratory values were eligible. Those with Division of AIDS (DAIDS) grade 1 (2.5 × upper limit of normal [ULN]) or higher transaminase levels; those who had used immunomodulatory drugs within 30 days of study start or concomitant medications likely to significantly reduce plasma levels of study drugs; and women who were pregnant/breastfeeding were excluded. Trial participation was voluntary and written informed consent was obtained from all subjects. The protocol, informed consent, and subject information form were reviewed and approved by the local Institutional Review Board (IRB)/Independent Ethics Committee (IEC) listed in S1 Appendix. The CONSORT check list is presented as S2 Appendix. The trial was approved by each site’s local Ethics Committee, before the start of the trial, and any amendments were approved during the conduct of the trial. The authors confirm that all related Phase 2–4 trials for tipranavir are registered. There are no longer any ongoing trials being conducted by the Sponsor.

### Study design

This study was a multinational, multicenter trial conducted from March 2, 2004, to June 22, 2006, at 74 sites in Western and Eastern Europe, Canada, Australia, Latin America, and Thailand. Patients were randomized to receive open-label TPV/r 500/100 mg BID, TPV/r 500/200 mg BID, or LPV/r 400/100 mg BID in combination with a backbone regimen of tenofovir disoproxil fumarate (TDF) and lamivudine (3TC). Patients were randomized centrally at a ratio of 1:1:1 in blocks of six. Centers were restricted to no more than 40 patients. Randomization was stratified by screening CD4 count (≤200 or >200 cells/mm^3^) but not by the center.

The primary efficacy endpoint was treatment response at week 48, defined as a confirmed virologic response (two consecutive VL measurements ≤50 copies/mL) without prior virologic failure, discontinuation of study drug, loss to follow-up, introduction of a new drug, or death. Secondary efficacy endpoints included time to treatment failure (TTF), using VL <50 copies/mL and VL <400 copies/mL as response criteria; treatment and virologic response at all visits, using VL <50 copies/mL and VL <400 copies/mL as response criteria; change from baseline in VL (log_10_ transformed) and CD4+ cell counts at each visit; patients with new Centers for Disease Control (CDC) AIDS-defining illness or death; and time to new AIDS-defining illness or death. Treatment failure is the complementary outcome to treatment success: the absence of confirmed virologic response (two consecutive VL measurements, ≤50 copies/mL) without prior virologic failure by week 48; discontinuation of study drug; loss to follow-up; introduction of a new ARV drug (except for reason of toxicity); or death. An additional analysis of TTF used ≤400 copies/mL as an alternative viral load threshold.

Safety endpoints were incidences of any adverse event (AE), any serious adverse events (SAEs), or chemistry and hematology test abnormalities. Analysis included empirical distribution of time to AEs and laboratory abnormalities of interest, including first SAE, grade 3/4 alanine aminotransferase (ALT) elevation, grade 3/4 aspartate aminotransferase (AST) elevation, occurrence of diarrhea and/or vomiting, grade 3/4 triglycerides abnormality, and grade 3/4 cholesterol abnormality. Provisions were made for treatment interruption to reduce transaminase elevations in cases with grade ≥3 transaminase elevations.

### Treatment

Patient and investigator were blinded to treatment assignment until after all eligibility criteria were met and the patient was randomized to his/her treatment assignment. Patients randomized to the TPV/r200 group initially took TPV/r 500/100 mg BID for 2 weeks. The 2-week lead-in regimen was used because the pre-steady state concentrations of TPV and RTV are higher in patients without prior liver enzyme induction (e.g., treatment-naïve patients) than is seen in patients with previous induction experience (e.g., treatment-experienced patients). This strategy aimed at avoiding TPV concentrations above the target level during the early dosing period in the 200 mg RTV group.

A backbone ARV regimen consisting of a nucleotide reverse transcriptase inhibitor (NtRTI, TDF 300 mg) plus a nucleoside reverse transcriptase inhibitor (NRTI, 3TC 300 mg) once daily was administered. A change from 3TC or TDF to another NRTI was allowed if toxicity or intolerance was clearly attributed to backbone medication. Patients were considered for withdrawal from the study and appropriate alternative therapy initiated if (i) VL had not dropped for at least 0.5 log_10_ after the initial 12 weeks of treatment; and (ii) patients failed to achieve VL < 100,000 copies/mL after 12 weeks of treatment, despite a 0.5 log_10_ copies/mL reduction after 12 weeks; or (iii) had VL > 400 copies/mL confirmed at two consecutive visits >2 weeks apart following a confirmed virologic response.

### Sample analysis

Plasma HIV-RNA concentrations, CD4+ cell counts, and safety laboratory evaluations were performed for every visit while patients remained on study. Study visits were scheduled at 2, 4, 12, 24, 36, and 48 weeks and then every 12 weeks until study completion. HIV-RNA concentrations were assayed and quantified using the Roche Amplicor^®^ HIV-1 Monitor Standard or Ultrasensitive Assay, Version 1.5, by Covance Central Laboratory Services. Baseline HIV genotypic resistance assessments were performed by VIRCO using their Virtual Phenotype^TM^ assay. Phenotypes were determined using VIRCO’s Antivirogram assay.

### Statistical analysis

The sample size was estimated using nQuery Advisor^®^ 4.0 by using the simulation of the lower confidence limit for difference in proportions for a one-sided 98.8% confidence interval (CI). A sample size of 180 per group was needed to achieve an 80% power for response rate in all groups of 70%. Primary efficacy analysis of available data was performed after all patients completed week 48. Analyses were based on treatment assigned at randomization. Primary analysis compared the treatment response of each TPV/r treatment group to that of the LPV/r group, using a two-sided 97.5% CI for the difference in response rates, adjusted by baseline CD4 strata (Bonferroni adjustment). Non-inferiority was concluded if the lower bound of the CI did not exceed the pre-defined non-inferiority margin of 15%. Additional analyses of the primary and secondary endpoints used descriptive statistics for all variables, logistic regression for treatment and virologic response, analysis of variance (ANOVA) for change from baseline in VL and CD4 count, and Cox proportional hazards and log-rank tests for time to event endpoints. Analysis of binary endpoints followed the intention-to-treat (ITT) principle, where missing values were replaced using the non-completers considered failures (NCF) approach. For continuous endpoints, including VL and CD4+ cell counts, change from baseline missing values was imputed for analysis using the last observation carried forward (LOCF) method. All analyses were adjusted for CD4 strata.

## Results

### Baseline patient characteristics

Demographics and patient baseline characteristics were similar between the three treatment groups (Table 1). Median age was 35 years (range, 18–74); population was predominantly male (76.5%) and white (73.3%). Median VL and CD4+ cell count for the ITT population were similar across treatment groups (overall median of 5.03 log_10_ copies/mL and 207 cells/mm^3^, respectively), as was rate of hepatitis B or C co-infection.

**Table 1:** Patient Demographic and Baseline Characteristics.

	Treatment group			
	TPV/r (500/100 mg)	TPV/r (500/200 mg)	LPV/r (400/100 mg)	Total
**Total treated (N)**	187	186	185	558
**Age (years)**				
Median (SD)	35.0 (9.3)	36.0 (10.4)	35.0 (10.2)	35.0 (10.0)
Range	18–71	18–65	18–74	18–74
**Gender**				
Male, n (%)	137 (73.3)	146 (78.5)	144 (77.8)	427 (76.5)
Female, n (%)	50 (26.7)	40 (21.5)	41 (22.2)	131 (23.5)
**Race**				
White, n (%)	141 (75.4)	134 (72.0)	134 (72.4)	409 (73.3)
Black, n (%)	20 (10.7)	22 (11.8)	27 (14.6)	69 (12.4)
Asian, n (%)	26 (13.9)	29 (15.6)	23 (12.4)	78 (14.0)
Missing, n (%)	0 (0.0)	1 (0.5)	1 (0.5)	2 (0.4)
**Baseline HIV RNA (log**_**10**_ **copies/mL)**				
N	187	185	185	557
Median	4.98	5.05	5.06	5.03
Range	3.40–6.73	3.43–6.50	3.53–6.87	3.40–6.87
**Non-B subtype**				
n (%)	50 (26.7)	51 (27.4)	56 (30.3)	157 (28.1)
**Baseline CD4+ counts (cells/mm**^**3**^**)**				
N	187	183	184	554
Median	216	197	207	207
Range	3.0–584	9.5–495	11.0–561	3.0–584
**Hepatitis co-infection**				
HBsAg and/or Anti-HCV +	32 (17.1)	34 (18.3)	31 (16.8)	97 (17.4)
HBsAg and/or Anti-HCV missing	2 (1.1)	4 (2.2)	3 (1.6)	9 (1.6)

BID = twice daily, HBsAg = hepatitis B surface antigen, HCV = hepatitis C virus, HIV = human immunodeficiency virus, LPV/r = lopinavir/ritonavir, n = number of evaluable subjects, SD = standard deviation, TPV/r = tipranavir/ritonavir.

AIDS-defining illnesses, based on CDC classifications (Categories C1–C3), had occurred in 11.1% of patients prior to enrollment.

### Baseline resistance characteristics and HIV-1 subtypes

Baseline genotyping and clade determination were performed in 535/562 randomized patients, of whom 240/535 (44.9%) patients had no TPV score mutations (L10V, I13V, K20M/R/V, L33F, E35G, M36I, K43T, M46L, I47V, I54 A/M/V, Q58E, H69K, T74P, V82L/T, N83D, and I84V) [32]. Of those patients who had TPV score mutations present at baseline, 71.2% had one or two, 24.7% had three, and 4.1% had four TPV score mutations. No patients had more than four TPV score mutations at baseline. No patients had reverse transcriptase mutations associated with the use of 3TC or TDF at baseline. Two patients had non-nucleoside reverse transcriptase inhibitor (NNRTI) resistance mutations at baseline: K103N and V106I (a minor etravirine mutation) [33]. The predominant HIV-1 viral subtype found in the patients in this study was clade B (378/535 [70.7%]). There were 157 patients (29.3%) infected with non-B subtype viruses, including subtype subtype F or BF, 8.6%; A/E mosaic (8.4%), subtype A (4.3%), subtype C or C recombinant (4.3%) and subtype G (including AG or BG), 3.0%. A greater proportion of non-B clade viruses at baseline had higher TPV mutation scores.

### Patient disposition

**Fig 1:**
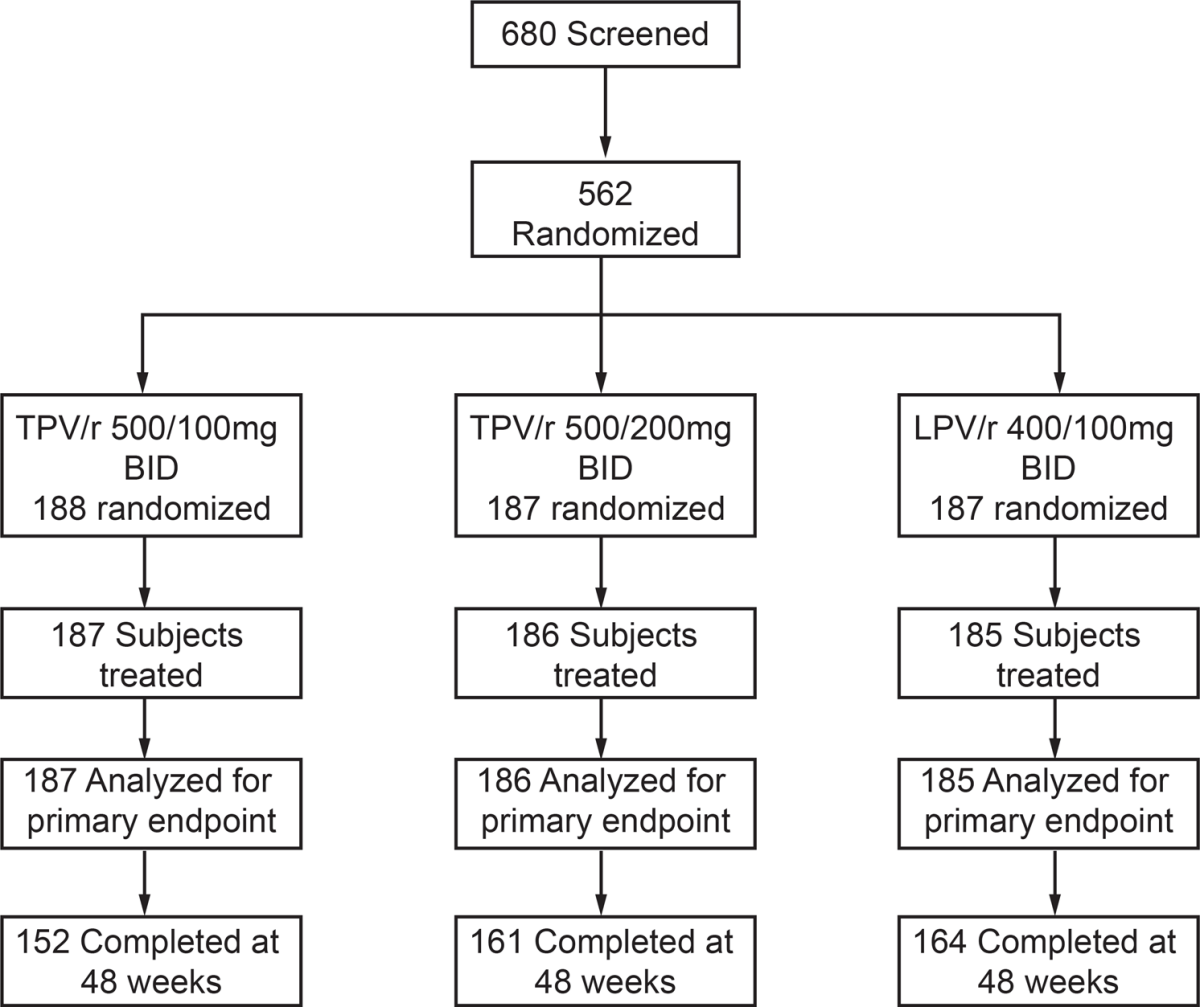
Patient disposition up to study closure.

Fig 1 shows the patient disposition. In total, 562 subjects were eligible and randomized; of these, four were not treated and three randomized to TPV/r100 but were treated with TPV/r200. In total, 426/558 (76.3%) treated patients completed 48 weeks. Further, 132 (23.7%) subjects prematurely discontinued therapy: 52/132 (39.4%) from the TPV/r100 group, 49/132 (37.1%) from the TPV/r200 group, and 21/132 (15.9%) from the LPV/r group. The primary cause of patient discontinuation was AEs (9.1%) followed by loss to follow-up (5.0%), withdrawn consent (3.0%), and virologic failure (1.8%). More patients in the TPV/r groups (9.8% for the TPV/r100 and 14.8% for TPV/r200) than in the LPV/r group (2.7%) discontinued due to AEs. Efficacy analyses included all 558 patients who were randomized and received at least one dose of study medication.

### Primary efficacy endpoint: Treatment response

Overall treatment response rates at week 48 were 68.4% (128/187) for the TPV/r100 group, 69.9% (130/186) for the TPV/r200 group, and 72.4% (134/185) for the LPV/r group. The CD4 strata-adjusted differences in response rates were −4.1 (97.5% CI: −14.5, 6.3) and −1.0 (97.5% CI: −11.4, 9.4) for TPV/r100 and TPV/r200, respectively, versus LPV/r.

**Fig 2:**
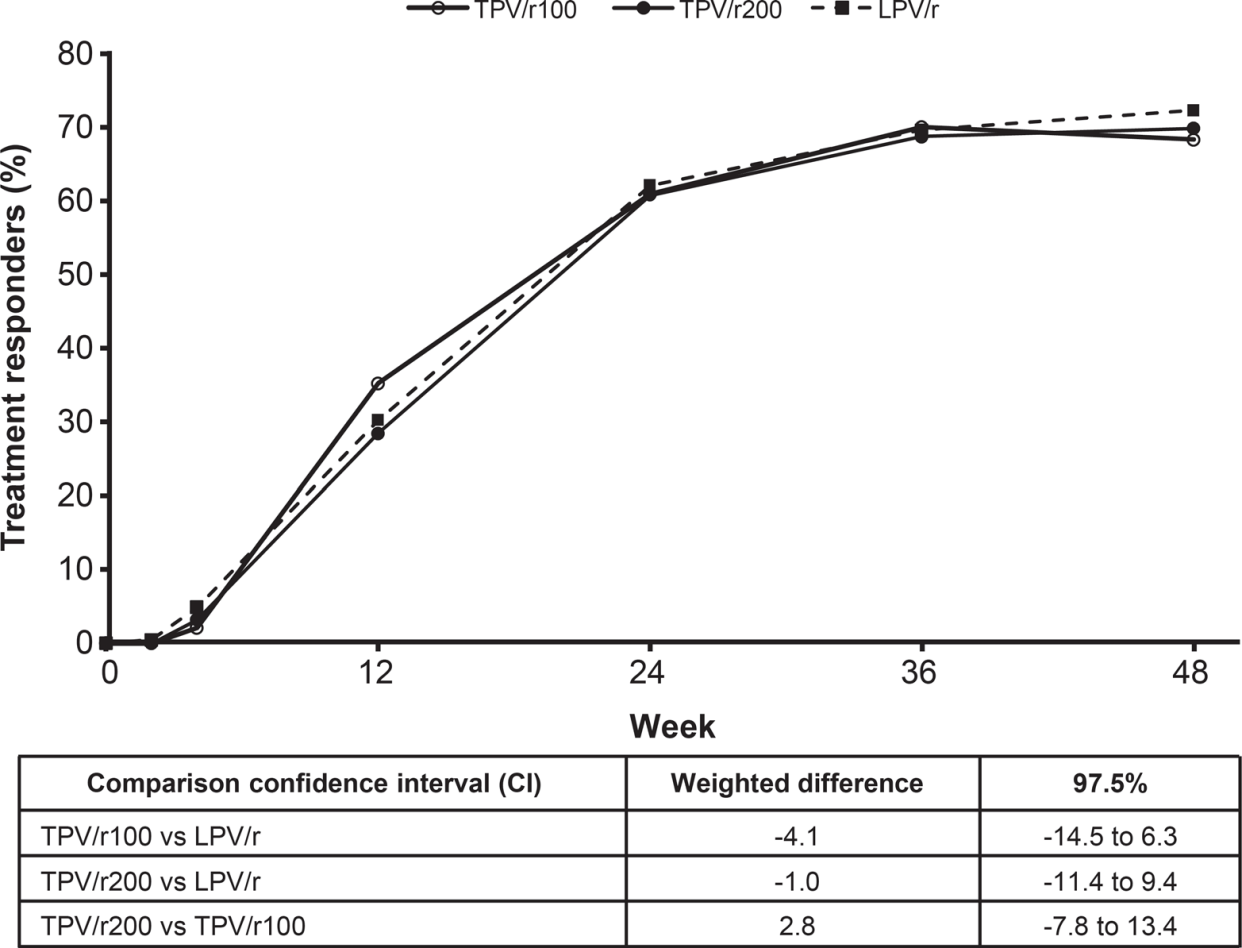
Treatment response up to week 48 (confirmed VL <50 copies/mL).

For the final, full dataset, both the TPV/r100 and TPV/r200 groups achieved non-inferiority to the LPV/r group at 48 weeks (Table 2, Fig 2). Proportions with treatment response were 68.4%, 69.9% and 72.4% for the TPV/r100, TPV/r200 and LPV/r groups, respectively.

**Table 2:** Outcome at Week 48 and Reasons for Failure of the Primary Endpoint–(As Randomized, Non-completers Considered Failures).

	Treatment group			
	TPV/r (500/100 mg), n (%)	TPV/r (500/200 mg), n (%)	LPV/r (400/100 mg), n (%)	Total, n (%)
**Total evaluable**	187 (100)	186 (100)	185 (100)	558 (100)
**Treatment response**	128 (68.4)	130 (69.9)	134 (72.4)	392 (70.3)
**Virologic failure**	30 (16.0)	27 (14.5)	28 (15.1)	85 (15.2)
VL <50 copies/mL without subsequent confirmation^a^	10 (5.3)	7 (3.8)	12 (6.5)	29 (5.2)
Never suppressed^b^	12 (6.4)	17 (9.1)	12 (6.5)	41 (7.3)
Rebound^c^	4 (2.1)	3 (1.6)	3 (1.6)	10 (1.8)
Drug changed or discontinued due to virologic failure^d^	4 (2.1)	0 (0.0)	1 (0.5)	5 (0.9)
**Death**^**e**^	1 (0.5)	0 (0.0)	1 (0.5)	2 (0.4)
**Study drug discontinued**	28 (15.0)	29 (15.6)	22 (11.9)	79 (14.2)
Adverse events	13 (7.0)	18 (9.7)	5 (2.7)	36 (6.5)
Other reasons	15 (8.0)	11 (5.9)	17 (9.2)	43 (7.7)

^a^Last viral load <50 copies/mL, without a subsequent confirming viral load <50 copies/mL

^b^Viral load never was suppressed to <50 copies/mL

^c^Confirmed loss of virologic response to >50 copies/mL or loss of virologic response and missing confirmatory visit, by week 48

^d^Includes premature discontinuation of the study protease inhibitor due to virologic failure and/or the addition of a drug to the backbone regimen (not discontinued due to an adverse event attributable to the backbone drug)

^e^Includes those events with fatal outcome with onset during the 48 week primary analysis treatment period

BID = twice daily, LPV/r = lopinavir/ritonavir, TPV/r = tipranavir/ritonavir, VL = viral load (plasma HIV-1 RNA).

In the low CD4 stratum (≤200 CD4 cells/mm^3^) for VL <50 copies/mL, treatment response rates at 48 weeks were 60.7% (51/84), 61.9% (52/84), and 62.0% (62/100) of patients in the TPV/r100, TPV/r200, and LPV/r groups, respectively. Differences in low CD4 stratum response rates were −1.2 (97.5% CI: −18.2, 15.8) and 0.1 (97.5% CI: −16.2, 16.4) for TPV/r100 and TPV/r200, respectively, versus LPV/r. The proportions of patients who achieved a treatment response at 48 weeks were greater in the high CD4 stratum (>200 CD4 cells/mm^3^) than the low CD4 stratum: 74.8% (77/103), 79.1% (68/86), and 81.2% (82/101) of patients in the TPV/r100, TPV/r200, and LPV/r groups, respectively. The differences in high CD4 stratum response rates were −6.4 (97.5% CI: −19.5, 6.6) and −2.1 (97.5% CI: −15.8, 11.6) for TPV/r100 and TPV/r200, respectively, versus LPV/r. The difference in response rates across strata can be attributed to a higher rate of virologic failures in the low CD4 strata (22.8%) than in the high strata (8.3%), with no notable differences among treatment groups.

Treatment response at week 48, analyzed using logistic regression and adjusted for CD4 stratum, indicated no significant difference in achieving a response across the treatment groups (odds ratios [ORs] of 0.82 [CI: 0.52, 1.29; p = 0.3824] and 0.94 [CI: 0.60, 1.49; p = 0.8011] for TPV/r100 and TPV/r200, respectively, versus LPV/r; overall p = 0.6648). CD4+ cell count at baseline had a significant effect on treatment response in all treatment groups, with patients in the low strata less likely to achieve a response than patients in the high strata (OR = 0.44 [CI: 0.30, 0.64]; p<0.0001). Participants entering the trial with baseline VL <100,000 copies/mL demonstrated similar treatment responses (<50 copies/mL) at 48 weeks, regardless of the treatment group (79.2%, 79.5%, and 81.2% for TPV/r100, TPV/r200, and LPV/r, respectively). The response rate for patients with baseline VL ≥100,000 copies/mL was less for all three treatment groups, with that for TPV/r100 being slightly lower than for other groups (56.7%, 62.7%, and 65.0% for TPV/r100, TPV/r200, and LPV/r, respectively). Following the primary endpoint and the decision to discontinue the experimental therapy, there was a substantial decrease in the number of patients who remained on assigned therapy (week 72, 71% [396/558]; week 84, 56% [315/558]), rendering subsequent data difficult to interpret.

Treatment failure was predominately due to virologic failure (lack of confirmed VL <50 copies/mL) and discontinuation due to AEs (Table 2). Virologic failure rates were comparable between treatment groups: 16.0% (30/187), 14.5% (27/186), and 15.1% (28/185) of patients in the TPV/r100, TPV/r200, and LPV/r groups, respectively. Treatment failure due to AE-related discontinuations was higher in the TPV/r groups than in the LPV/r group: 7.0% (13/187), 9.7% (18/186), and 2.7% (5/185) of patients in the TPV/r100, TPV/r200, and LPV/r groups, respectively.

### Secondary efficacy endpoints

TTF using a virologic threshold for failure of <50 copies/mL at 48 weeks was analyzed using a Cox proportional hazards model, adjusting for CD4 stratum. Distributions of TTF were statistically different when comparing either TPV/r group to LPV/r, with Cox proportional hazards p-values of 0.0017 (HR = 1.74 [95% CI: 1.39, 2.09]) and 0.0014 (HR = 1.81 [95% CI: 1.44, 2.18]) for TPV/r100 and TPV/r200, respectively, versus LPV. Similar results were observed using <400 copies/mL as the threshold.

Virologic response rates, using VL <50 copies/mL (ITT-NCF analyses; Fig 2), VL <400 copies/mL, and 1 log_10_ drop in VL, were comparable across the groups. VL reduction from baseline was rapid and sustained across all treatment groups (ITT-LOCF analyses). VL reductions from baseline were sustained to week 48, with median changes from baseline at week 48 of −3.10 (interquartile range [IQR]: −2.70, −3.49), −3.15 (IQR: −2.67, −3.56), and −3.27 (IQR: −2.77, −3.62) log_10_ copies/mL for TPV/r100, TPV/r200, and LPV/r, respectively. ANOVA results for differences in VL at week 48 were not statistically significant between TPV/r100 and LPV/r (0.136; 95% CI: −0.045, 0.317; p = 0.1412) and between TPV/r200 and LPV/r (0.117; 95% CI: −0.065, 0.298; p = 0.2088).

Immunological improvement from baseline was evident early in all treatment groups (ITT-LOCF), with median increases of 55 (IQR: 15, 99), 52 (IQR: 20, 101), and 57 (IQR: 15, 99) cells/mm^3^ for TPV/r100, TPV/r200, and LPV/r, respectively, at week 2. Median increases in CD4 cell count at week 48 were 172 (IQR: 104, 266), 175 (IQR: 95, 263), and 207 (IQR: 111, 275) cells/mm^3^ for the TPV/r100, TPV/r200, and LPV/r groups, respectively. ANOVA results for differences in CD4 count at week 48 were not statistically significant between TPV/r100 and LPV/r (−19.42; 95% CI: −46.16, 7.322; p = 0.1543) and between TPV/r200 and LPV/r (−12.47; 95% CI: −39.41, 14.46; p = 0.3634).

The number of patients who experienced AIDS-defining illness or death was similar among all three groups (four in the LPV/r group, five in the TPV/r200 group, and eight in the TPV/r100 group). The time to a treatment-emergent AIDS-defining illness or death was not significantly different among treatment groups (Cox regression: p = 0.2176 comparing TPV/r100 versus LPV/r; p = 0.5024 comparing TPV/r200 versus LPV/r). Of these events, there were 2, 1, and 5 deaths in the LPV/r, TPV/r200, and TPV/r100 groups, respectively. No deaths were related to study medication, and no particular AIDS-defining illness predominated: PCP (TPV/r100, 3 subjects), HSV (TPV/r200, 1; LPV/r, 1), tuberculosis (TPV/r200, 1; LPV/r, 1), and 1 each with Kaposi’s sarcoma, leukoencephalopathy, immunoblastoma, lymphoma, recurrent pneumonia, and wasting syndrome.

There was no evidence of reduced responses to TPV/r in patients infected with non-clade B virus.

### Resistance emergence evaluation

A subset of all 21 patients with virologic failure or rebound during study treatment underwent on-treatment resistance testing to characterize the emergence of protease gene and reverse transcriptase mutations. Evaluation of resistance patterns was determined using samples from those patients with virologic failure by week 24 (nine patients from the TPV/r100 group, nine from the TPV/r200 group, and three from the LPV/r group). Three TPV/r patients (one TPV/r100: Q58E, two TPV/r200: I84V and I13V) developed a single mutation associated with TPV during the study. Based on TPV half-maximal inhibitory concentration (IC_50_), the virus for all three patients remained fully susceptible to TPV (≤3-fold change in IC_50_ compared to wild-type control virus). There was no change in protease inhibitor (PI) phenotypic resistance classification for any of these 21 patients.

Only the M184I/V mutation emerged as a reverse transcriptase mutation associated with resistance to 3TC and TDF. There were 11/21 (52.4%) patients who displayed this mutation at the time of treatment failure.

### Safety

Safety was assessed in all 558 patients who received at least one dose of study medication. Proportions of patients who experienced at least one AE while on treatment were similar among the three groups. SAEs and AEs leading to discontinuation tended to be more frequent among TPV/r groups (Table 3).

**Table 3:** Frequency of Patients with Adverse Events and Grade 3/4 Laboratory Abnormalities–All Randomized Patients Who Took at Least One Dose of Study Medication.

	Treatment Group		
	TPV/r (500/100 mg BID), n (%)	TPV/r (500/200 mg BID), n (%)	LPV/r (400/100 mg BID) n (%)
**Patients treated**	184 (100%)	189 (100%)	185 (100%)
**Patients with any AE**	164 (89.1%)	175 (92.6%)	171 (92.4%)
Diarrhea	94 (51.1%)	97 (51.3%)	98 (53.0%)
Nausea	72 (39.1%)	76 (40.2%)	48 (25.9%)
Headache	35 (19.0%)	25 (13.2%)	30 (16.2%)
Nasopharyngitis	32 (17.4%)	25 (13.2%)	29 (15.7%)
Vomiting	29 (15.8%)	19 (10.1%)	17 (9.2%)
Abdominal pain	23 (12.5%)	27 (14.3%)	16 (8.6%)
**Grade 3/4 laboratory abnormalities**			
Cholesterol^a^	11 (6.0%)	13 (7.0%)	5 (2.7%)
Triglycerides^a^	14 (7.6%)	9 (4.8%)	10 (5.5%)
Alanine aminotransferase^a^^,^^b^	13 (7.1%)	41 (21.9%)	8 (4.4%)
Aspartate aminotransferase^a^^,^^b^	6 (3.3%)	19 (10.2%)	8 (4.4%)
**Any drug-related AE**	121 (65.8%)	134 (70.9%)	111 (60.0%)
**Any AE leading to discontinuation**	19 (10.3%)	29 (15.3%)	6 (3.2%)
**Any serious AE**	26 (14.1%)	26 (13.8%)	16 (8.6%)
**Deaths**^**c**^	5 (2.7%)	2 (1.1%)	2 (1.1%)

^a^Percentages of patients with laboratory assays performed: TPV/r100 (n = 183); TPV/r200 (n = 187); LPV/r (n = 182)

^b^After continued treatment or following re-introduction after treatment interruption, transaminase elevations returned to grade ≤2 in more than 65% of patients receiving either TPV/r200 or TPV/r100

^c^Includes those events with fatal outcome with onset during the 48 week primary analysis treatment period and the post-48 week follow-up treatment

AE = adverse event, BID = twice daily, LPV/r = lopinavir/ritonavir, TPV/r = tipranavir/ritonavir.

The most frequently observed AEs across the three groups were diarrhea, nausea, headache, nasopharyngitis, abdominal pain, and vomiting; however, gastrointestinal symptoms and grade 3 or 4 plasma cholesterol were observed more frequently in the TVP/r groups (Table 3). Overall, 72.8%, 75.7%, and 70.8% of TPV/r100, TPV/r200, and LPV/r patients, respectively, experienced gastrointestinal (GI) AEs, few of which were severe in intensity: 2.2%, 3.7%, and 2.7% for TPV/r100, TPV/r200, and LPV/r, respectively (Table 4). Differences in discontinuations due to AEs (19, 29, and 6 for TPV/r100, TPV/r200, and LPV/r, respectively) are largely due to more TPV/r than LPV/r patients discontinuing due to GI AEs (7, 8, and 1 patients, respectively) and ALT/AST elevations (5, 14, and 2 patients, respectively) (Table 4).

**Table 4:** Frequency of Patients with Gastrointestinal Adverse Events by Severity and Treatment Discontinuation, and Liver Transaminase Elevations Leading to Discontinuation.

	Treatment Group		
	TPV/r (500/100 mg BID)	TPV/r (500/200 mg BID)	LPV/r (400/100 mg BID)
**Patients treated, N (%)**	184 (100)	189 (100)	185 (100)
**Patients with GI-related AE, n (%)**	134 (72.8)	143 (75.7)	131 (70.8)
Mild	76 (41.3)	83 (43.9)	82 (44.3)
Moderate	54 (29.3)	53 (28.0)	44 (23.8)
Severe	4 (2.2)	7 (3.7)	5 (2.7)
**Patients with any AEs leading to discontinuation, n (%)**	19 (10.3)	29 (15.3)	6 (3.2)
**Patients with discontinuation due to GI AEs**^a^	7	8	1
Mild	1	2	0
Moderate	5	4	1
Severe	1	2	0
**Patients with discontinuation due to ALT/AST elevations**^a^	5	14	2
Mild	0	2	0
Moderate	3	3	0
Severe	2	9	2

^a^Persistence of transaminase elevations despite treatment interruption

AE = adverse event, ALT = alanine aminotransferase, AST = aspartate aminotransferase, BID = twice daily, GI = gastrointestinal, LPV/r = lopinavir/ritonavir, TPV/r = tipranavir/ritonavir.

Sixty-eight (12.2%) patients experienced SAEs by week 48. The overall rate was similar for the TPV/r100 group and TPV/r200 group (26 [14.1%] and 26 [13.8%] patients, respectively) and tended to be lower for the LPV/r group (16 [8.6%] patients). However, the rate of drug-related SAEs was comparable among treatment groups (two reported for each group).

Patients in the TPV/r200 group had a higher incidence of grade 3/4 ALT elevations (21.9%) compared to those in the TPV/r100 group (7.1%) and the LPV/r group (4.4%) (Table 3). Kaplan-Meier estimates of time to first grade 3/4 ALT or AST elevation were higher in the TPV/r200 group than the other two treatment groups: Grade 3/4 ALT at week 48, approximately 15% for TPV/r200, 6% for TPV/r100, and 4% for LPV/r; for grade 3/4 AST at week 48, approximately 8%, 3%, and 2.5%, respectively (log Rank p<0.0001 for ALT and p = 0.0061 for AST compared to LPV/r). Elevated ALT or AST values at baseline and active hepatitis B or C co-infection at baseline were associated with greater frequency of on-treatment grade 3/4 ALT or AST levels for all three groups. ALT levels returned to grade ≤2 in all but one patient in the TPV/r100 group, all but two patients in the TPV/r200 group, and all but two patients in the LPV/r group. ALT levels remained stable at grade 3/4 or increased after TPV/r discontinuation in one of these patients in each of the TPV/r200 and LPV/r groups. AST levels returned to grade ≤2 in all patients in the TPV/r100 group, all but one patient in the TPV/r200 group, and all but two patients in the LPV/r group. AST levels remained stable or increased after TPV/r discontinuation in one of these patients in each of the TPV/r200 and LPV/r groups. Treatment interruptions and discontinuations as a result of grade 3/4 ALT and AST elevations were most common in the TPV/r200 group (22/41 [53.7%] and 12/19 [63.2%] patients with ALT and AST elevations, respectively) and occurred at a higher frequency than in treatment-experienced patients [2]. However, upon continued treatment or following re-introduction after treatment interruption, transaminase elevations returned to grade ≤2 in more than 65% of patients receiving either TPV/r200 or TPV/r100. There were no clinical hepatic events reported in any of the trial patients, including those with ALT/AST elevations.

Incidences of grade 3 lipid abnormalities were similar across the groups and lower than in previous TPV trials with treatment-experienced patients, with the exception of cholesterol in the TPV/r200 group [1, 2, 31]. Grade 3 total cholesterol elevations were reported in 7.0%, 6.0%, and 2.7% of patients in the TPV/r200, TPV/r100, and LPV/r groups, respectively. The Kaplan-Meier estimate of time to first grade 3 cholesterol abnormality was higher in the TPV/r200 group compared to LPV/r (Log Rank p = 0.0300). There were no grade 4 elevations. Grade 3 or 4 triglyceride elevations were similar across treatment groups: 7.6% of patients in the TPV/r100 group, 4.8% in the TPV/r200 group, and 5.5% in the LPV/r group. The Kaplan-Meier estimates of time to first grade 3 or 4 triglyceride abnormalities showed no significant differences between the TPV/r groups and the LPV/r group.

Other laboratory abnormalities were generally grade 1 or 2 and occurred with similar frequencies among the three groups.

Based on the higher frequency of liver transaminase elevations in the TPV/r200 group, the external Data Safety Monitoring Board recommended closure of this treatment group during the post-48-week follow-up period of the trial. Since the primary objectives had been achieved and continuing TPV/r100 was perceived as being less tolerable than standard of care for initial cART, the trial was discontinued during the post-48-week follow-up, with 436 (76.3%) patients (132 [71.7%], 140 [74.1%], and 164 [88.6%] patients in the TPV/r100, TPV/r200, and LPV/r groups, respectively) in the trial at that time.

## Discussion

Trial 1182.33 explored the effectiveness and safety of TPV/r in treatment-naïve patients, using the dose employed for HIV-1-infected patients harboring PI-resistant virus, along with a lower investigational exposure to TPV, as a result of a lower dose of RTV (100 mg). Overall, the results demonstrated that 500 mg TPV, administered with either 100 mg or 200 mg RTV, achieved substantial VL reduction to undetectable levels and immunologic responses in HIV-1-infected treatment-naive patients. Treatment response (confirmed VL ≤50 copies/mL) evaluated at 48 weeks showed that both the TPV/r100 and TPV/r200 were non-inferior to LPV/r, based on a non-inferiority margin of 15%. Additionally, reduction in VL was rapid for all three treatment groups, with between −1.5 and −2 log_10_ median decreases at 2 weeks. At 48 weeks, similar VL reductions (greater than 3 log_10_ copies/mL) were observed for all three treatment groups. Immunological recovery was evident in TPV and LPV groups, with sustained increases in CD4+ cell count. These results support the potent antiviral effect of TPV. These final results differ slightly from the preliminary results previously presented [34], because missing data on one patient in the TPV/r100 group became available only after the Glasgow presentation. At the time, it appeared that the TPV/r100 group exceeded the 15% boundary for non-inferiority (treatment response for LPV/r: 72.4%; TPV/r100: 67.9%; [difference: −4.6% (−15.03, 5.8)]). The response rates for TPV/r200 and LPV/r remain unchanged between the Glasgow meeting and the final analyses ((treatment response for TPV/r200: 69.9%; [difference: −1.0% (−11.4, 9.4)]).

It should be noted that since this trial was intended as the initial examination of TPV/r in naïve patients, the sample size was modest and therefore the non-inferiority bound was set at 15%, since this was considered an exploratory Phase 2B trial.

Subset analysis using the baseline stratification of patients by CD4+ cell count and VL showed similar results between treatment groups. As seen in studies of other treatment-naïve patients, patients in the higher CD4+ strata were more likely to achieve treatment responses [35].

HIV genotypes were mostly of clade B subtype, but the proportion of non-B subtypes with three or four TPV score mutations was almost 40-fold greater than that of the B subtype at baseline. Furthermore, the distribution of non-B subtypes was similar across treatment groups. The Stanford University HIV Resistance Database shows that non-B subtypes tend to be more TPV resistant [36]. Despite patients with non-B subtypes tending to have more TPV score mutations at baseline, there was no evidence of meaningful response differences between clade B and non-B subtypes.

The protease inhibitor polymorphisms detected at trial entry represent PI non-key mutations; these polymorphisms differ from the key mutations, the presence of which results in high level PI resistance. These polymorphisms almost certainly represent naturally occurring sequences and are unlikely to imply any prior PI treatment in the study’s treatment naïve patients.

Testing of the post treatment isolates revealed no emergent phenotypic resistance upon virologic rebound. Specifically, the phenotypic susceptibility classification did not change for any of the 21 patients whose post-rebound isolates were tested. Reverse transcriptase mutation M184I/V was identified in over half of the viruses from patients with virologic rebound, similar to that reported previously in studies of PIs used with 3TC [37]. Absence of any emerging PI resistance during treatment with TPV was expected due to the high genetic barrier to resistance, as has been seen with other RTV-boosted PIs in HIV-1-infected treatment-naïve patients.

Most commonly reported AEs among all three treatment groups were GI events, mostly moderate in intensity. Compared to the LPV/r group, both TPV/r groups had higher discontinuations rates due to AEs, largely due to higher rates of discontinuation for GI AEs and transaminase elevations in both TPV/r arms. The open-label design could have influenced investigators’ decisions to discontinue therapy in TPV patients with GI events. However, a majority of TPV/r200 patients with grade 3/4 transaminase increases could continue therapy uninterrupted or after re-challenge, and levels returned to grade ≤2 in all instances. Fatalities during the trial were uncommon among these treatment-naive patients; a total of nine (1.6%) deaths were reported, of which eight were during treatment or within 30 days following drug discontinuation. One occurred after 30 days of follow-up. Few episodes of treatment-emergent AIDS-defining illnesses were reported. There were only six drug-related SAEs reported. The results indicate that TPV meets the non-inferiority criteria used in this trial despite the reported AE rates, based on the predefined non-inferiority margin of 15% (which is larger than the current generally accepted margin of 10%–12%).

Finally, it should be noted that the results of this study suggest that TPV/r should not be considered for use in treatment-naïve HIV patients. Although the trial demonstrated efficacy similar to a standard of care at the time the trial was conducted, the safety and drug interaction profiles [38] are not compatible with first-line therapy. Furthermore, the current standards of care have evolved; neither the IAS-USA nor DHHS Guidelines now include LPV/r + TDF + 3TC as first-line therapy [4, 39]. Therefore, the first-line therapy standard to which TPV/r was compared is no longer as relevant as it was in 2004–2006. Currently recommended first-line therapy is generally recognized as having fewer drug-associated adverse events, a more manageable drug interaction profile and more patient-friendly pill burden. For second-line or later therapy, it is critical that new regimens contain at least 2 (preferably 3) active ARV drugs [39]. Therefore, there may be need for TPV/r-containing combination regimens in a small number of late-stage treatment patients, who have developed PI resistance, and are unable to use other regimens.

## Conclusions

In this trial, conducted in 2004–2006, both TPV/r100 and TPV/r200 regimens showed sustained treatment responses in approximately 70% of patients, which were non-inferior to LPV/r at 48 weeks. VL reductions and CD4+ cell count increases were similar among treatment groups. However, these TPV/r regimens do not appear to be appropriate options among currently available therapies for treatment-naïve patients. Current therapies have improved adverse event and drug interaction profiles, as well as more patient-friendly pill burdens. The authors suggest that TPV/r regimens should not be used for treatment-naïve patients.

## Supporting Information

S1 Appendix List of local Independent Ethics Committees and Institutional Review boards (DOCX)

S2 Appendix CONSORT check list (DOC)

## Abbreviations

ARVs: antiretroviral drugs
ALT: alanine aminotransferase
AST: aspartate aminotransferase
cART: combination antiretroviral therapy
CPI/r: comparator protease inhibitor
CDC: Centers for Disease Control
DAIDS: Division of AIDS
LPV/r: ritonavir-boosted lopinavir
3TC: lamivudine
NtRTI: nucleotide reverse transcriptase inhibitor
NRTI: nucleoside reverse transcriptase inhibitor
NNRTI: non-nucleoside reverse transcriptase inhibitor
NCF: non-completers considered failures
OBR: optimized backbone regimen
PI: protease inhibitor
RTV: ritonavir
TPV/r: ritonavir-boosted tipranavir
TDF: tenofovir disoproxil fumarate
TTF: time to treatment failure
VL: viral load
