# Supplementary material for: Tipranavir/Ritonavir (500/200 mg and 500/100 mg) Was Virologically Non-Inferior to Lopinavir/Ritonavir (400/100 mg) at Week 48 in Treatment-Naïve HIV-1-Infected Patients: A Randomized, Multinational, Multicenter Trial
Source: PLoS One. 2016 Jan 5;11(1):e0144917. doi: 10.1371/journal.pone.0144917 (PMC4701182; doi:10.1371/journal.pone.0144917)
Supplement: Supplementary file 2 [file pone.0144917.s001.docx]

Argentina IEC / Investigator					
Investigator/ Study Site	Independent Ethics Committee	IRB or EC Chairman	Date of Final Approval		
			Protocol	Amendment 1	Amendment 2
Dr. Pedro Cahn/ 5401	IEC "Dr. Virgilio G. Foglia"	Dr. Carlos A. Barclay	08 March 04	28 February 05	30 September 05
Dr. Isabel Cassetti/ 5402	IEC "Dr. Virgilio G. Foglia"	Dr. Carlos A. Barclay	08 March 04	28 February 05	30 September 05
Dr. Arnaldo Casiro / 5403	IEC "Dr. Virgilio G. Foglia"	Dr. Carlos A. Barclay	08 March 04	28 February 05	30 September 05
Dr. Héctor Laplumé / 5404	IEC "Dr. Virgilio G. Foglia"	Dr. Carlos A. Barclay	08 March 04	28 February 05	30 September 05

Australia IRBs / Investigator					
Investigator/ Study Site	IRB or Ethics Committee	IRB or EC Chairman	Date of Final Approval		
			Protocol	Amendment 1	Amendment 2
Cooper/6101	St Vincent's Hospital, Human Research Ethics Committee	Prof. Jo-Ann Brien	03 March 04	19 April 05	03 Mar 06
Hoy/6102	The Alfred Ethics Committee	R Frew	04 March 04	8 April 05	08 Dec 05
Workman/6103	South Eastern Sydney Area Health Service Human Research Ethics Committee	Kim Breheny	01 April 04	28 April 05	09 Dec 05
Baker/6105	St Vincent's Hospital, Human Research Ethics Committee	Prof. Jo-Ann Brien	03 March 04	19 April 05	03 Mar 06
Moore/6106	Royal Australian College of General Practioners	Prof. Nick Zwar	15 April 04	15 May 05	19 Dec 05

Australia IRBs / Investigator					
Investigator/ Study Site	IRB or Ethics Committee	IRB or EC Chairman	Date of Final Approval		
			Protocol	Amendment 1	Amendment 2
Roth/6107	Royal Australian College of General Practioners	Prof. Nick Zwar	15 April 04	15 May 05	19 Dec 05
Quin/6108	South Western Area Health Service Human Research Ethics Committee	Prof. Hugh Dickson	03 May 04	21 April 05	pending
Gold/6109	South Eastern Sydney Area Health Service Human Research Ethics Committee	Kim Breheny	07 May 04	23 March 05	10 Feb 06

Bahamas IRBs / Investigator					
Investigator/ Study Site	IRB or Ethics Committee	IRB or EC Chairman	Date of Final Approval		
			Protocol	Amendment 1	Amendment 2
Dr. Perry Gomez/11017	The Public Hospitals Authority Ethics Committee Nassau, Bahamas	Dr. Glen Beneby	26 Apr 2004	18 March 05	

Brazil IRBs / Investigator					
			Date of Final Approval		
**Investigator/ Study Site**	**IRB or Ethics Committee**	**IRB or EC Chairman**	**Protocol**	**Amendment 1**	**Amendment 2**
Dr. Adauto Castelo Filho / 5510	Universidade Federal de São Paulo – Hospital São Paulo	Dr. José Osmar Medina Pestana	27 April 04	29 March 05	03 November 05
Dr. José Luiz de Andrade Neto / 5514	Comitê de Ética em Pesquisa em seres Humanos - PUCPR	Dr. Emílio José Scheer Neto	07 April 04	07 April 05	11 November 05
Dr. José Valdez Madruga / 5505	Centro De Referência e Treinamento DST/AIDS	Eduardo Ronner Lagonegro	05 May 04	04 April 05	07 Novmeber 05
Dr. Artur Timerman / 5501	Comitê de Ética em Pesquisa Hospital Heliópólis	Jozias de Andrade Sobrinho	16 April 04	12 April 05	31 October 05
Dr. Beatriz Grinsztejn / 5502	Comitê de Ética em Pesquisa do IPEC	Dr. Léa Camillo Coura	12 April 04	06 June 05	13 December 05
Dr. Marília Santini / 5506	Comitê de Ética em Pesquisa do IPEC	Dr. Léa Camillo Coura	12 April 04	06 June 05	13 December 05

Brazil IRBs / Investigator					
			Date of Final Approval		
**Investigator/ Study Site**	**IRB or Ethics Committee**	**IRB or EC Chairman**	**Protocol**	**Amendment 1**	**Amendment 2**
Dr. Roberto Zajdenverg / 5508	Comitê de Ética em Pesquisa HUCFF/ UFRJ	Dr. Luis Carlos Duarte de Miranda	07 May 04	02 June 05	09 December 05

Canada IRBs / Investigator					
Investigator/ Study Site	IRB or Ethics Committee	IRB or EC Chairman	Date of Final Approval		
			Protocol	Amendment 1	Amendment 2
Dr. William Cameron/11001	Ottawa Hospital Research Ethics Board Ottawa, ON	Dr. Francine Sarazin	25 March 04	18 March 05	28 October 05
Dr. Brian Conway/11002	UBC-Providence Health Care Vancouver, BC	Dr. S. Shalansky	24 June 04	05 July 05	Pending
Dr. Pierre Cote/11003	Ethica Clinical Research Inc. Montreal, QC	Dr. Barry F. Brown	24 February 04	03 March 05	14 October 05
Dr. Kevin Gough/11004	St. Michael's Hospital Research Ethics Board Toronto, ON	Dr. C.A. Ottaway	29 March 04	27 May 05	20 January 06
Dr. Richard Lalonde/11006	McGill University Research Ethics Board Montreal, QC	Dr. Dick Menzies	04 May 04	26 April 05	3 November 05
Dr. Anita Rachlis/11007	Ethics Review Board Sunnybrook and Women's College Health Sciences Centre Toronto, ON	Dr. Philip Hébert	10 May 04	19 May 05	17 January 06

Canada IRBs / Investigator					
Investigator/ Study Site	IRB or Ethics Committee	IRB or EC Chairman	Date of Final Approval		
			Protocol	Amendment 1	Amendment 2
Dr. Stuart Rosser/11008	Biomedical Research Ethics Board Winnipeg, MN	Dr. Nicholas Anthonisen	15 April 04	24 February 05	14 November 05
Dr. Michael Silverman/11009	Lakeridge Health Research Ethics Board Oshawa, ON	Joseph Neal, BA, LL.B	05 April 04	16 May 05	8 November 05
Dr. Fiona Smaill/11010	Hamilton Health Sciences Research Ethics Board Hamilton, ON	Dr. F. Jack Holland	23 March 04	17 May 05	Pending
Dr. Michel Boissonnault /11011	IRB Services Aurora, ON	Dr. George Price	15 June 04	16 February 05	19 October 05
Dr. Chris Tsoukas/11012	McGill University Research Ethics Board Montreal, QC	Dr. Dick Menzies	21 May 04	15 March 05	Pending
Dr. Sharon Walmsley/11013	University Health Network Research Ethics Board Toronto, ON	Dr. Ronald Heslegrave	09 March 04	11 April 05	Pending

Canada IRBs / Investigator					
Investigator/ Study Site	IRB or Ethics Committee	IRB or EC Chairman	Date of Final Approval		
			Protocol	Amendment 1	Amendment 2
Dr. Frederic Crouzat/11018	IRB Services Aurora, ON	Dr. George Price	25 March 04	16 February 05	19 October 05

Columbia IRBs / Investigator					
Investigator/ Study Site	IRB or Ethics Committee	IRB or EC Chairman	Date of Final Approval		
			Protocol	Amendment 1	Amendment 2
Dr. Carlos Alvarez / 5701	ETICHS COMMITTEE – "COMITÉ DE INVESTIGACIONES Y ÉTICA HOSPITAL DE SAN IGNACIO"	Dr. Francisco Henao	30 April 04	16 June 05	12 October 05
Dr. Alvaro Arango / 5702	ETHICS COMMITTEE – "COMITÉ DE ÉTICA EN INVESTIGACIONES CLÍNICAS FUNDACIÓN CARDIO INFANTIL"	Dr. Sinay Arevalo	29 April 04	07 June 05	12 October 05
Dr. Ricardo Leal / 5703	ETHICS COMMITTEE – "COMITÉ DE ETICA CENTRO DE INVESTIGACIONES ONCOLÓGICAS"	Dr. Luis Enrique Florez	16 February 04	05 April 05	14 October 05

Columbia IRBs / Investigator					
Investigator/ Study Site	IRB or Ethics Committee	IRB or EC Chairman	Date of Final Approval		
			Protocol	Amendment 1	Amendment 2
Dr. Otto Sussmann / 5704	ETHICS COMMITTEE – "COMITÉ DE INVESTIGACIONES FUNDACIÓN ABOOD SHAIO"	Dr. Hernando Orjuela	05 April 04	23 May 05	25 October 05

FRANCE IRBs / Investigator					
Investigator/ Study Site	IRB or Ethics Committee	IRB or EC Chairman	Date of Final Approval		
			Protocol	Amendment 1	Amendment 2
**Same IRB for all investigators**	CCPPRB Montpellier St-Eloi<br>Hôpital Saint Eloi<br>34295 Montpellier Cedex 5	Pr. Sylvie HANSEL	06 April 04	01 March 05	15 November 05

Germany IRBs / Investigator					
Investigator/ Study Site	IRB or Ethics Committee	IRB or EC Chairman	Date of Final Approval		
			Protocol	Amendment 1	Amendment 2
All sites	Landesärztekammer Baden-Württemberg	Dr. med. Th. Aleker	07 January 04	08 February 05	29 September 05
Dr. Arastéh / 4901	Ärztekammer Berlin	Prof. Dr. Ruth Mattheis	15 January 04	23 February 05	29 September 05
Dr. Stoehr / 4916	Ärztekammer Hamburg	Prof. Dr. med. Thomas Weber	03 February 04	04 March 05	N/A
Dr. Mauss / 4914	Ärztekammer Nordrhein	Prof. Dr. med. Sehrt-Ricken	13 February 04	22 February 05	05 October 05
Dr. Bergmann / 4902	Ethik-Kommission der Charité Berlin	Prof. Dr. H. Eichstaedt	16 February 04	10 March 05	24 October 05
Dr. Oette / 4912	Ethik-Kommission der Universität Düsseldorf	Prof. Dr. H.-G. Lenard	01 June 04	01 March 05	
Dr. Esser / 4904	Ethik-Kommission der Universität Essen	Prof. Dr. Karl H. Jakobs	24 March 04	03 March 05	28 October 05

Germany IRBs / Investigator					
Investigator/ Study Site	IRB or Ethics Committee	IRB or EC Chairman	Date of Final Approval		
			Protocol	Amendment 1	Amendment 2
Prof. Goebel / 4907	Ethik-Kommission der Ludwig-Maximilians Universität München	Prof. Dr. Gustav Paumgartner	01 March 04	09 May 05	25 Apr 06
Prof. Fätkenheuer / 4905	Ethik-Kommission der Universität Köln	Prof. Dr. med. M. Staak	11 March 04	08 June 05	09 Mar 06

MEXICO IRBs / Investigator					
Investigator/ Study Site	IRB or Ethics Committee	IRB or EC Chairman	Date of Final Approval		
			Protocol	Amendment 1	Amendment 2
Mario Jauregui / 5231	Ethics Committee of Hospital Regional "LIC Adolfo López Mateos" ISSST Mexico DF	Hector Hurtado Reynaector Hurtado Reyna	01 April 04	06 June 05	09 March 06
Eduardo Zapata / 5232	Ethics Committee of Hospital Regional de Especialidaes #25 CMNE IMSS Monterrey Nuevo Leon Mexico	Pablo Moreno Guevara	03 May 04	May 05	27 March 06
Paulo López / 5233	Ethics Committee of Hospital Regional # 25 IMSS Guadalajara Jalisco Mexico	German López Guillen	19 May 04	29 Apr 05	November 05

POLAND IRBs / Investigator					
Investigator/ Study Site	IRB or Ethics Committee	IRB or EC Chairman	Date of Final Approval		
			Protocol	Amendment 1	Amendment 2
Prof. Andrzej Gładysz / 4801	Komisja Bioetyczna 50-367 Wrocław	Prof. Franciszek Iwańczak	10 March 04	10 March 05	submitted on 19 Oct 05 with stamp of 25 Oct 05 (silent approval)
Dr Marek Beniowski /4803	Komisja Bioetyczna Śląskiej Izby Lekarskiej 40-126 Katowice	Dr hab. Krystyn Sosada	26 April 04	28 Feb 05	submitted on the 19 Oct 05 with stamp of 21 Oct 05 (silent approval)
Prof. Anna Boroń-Kaczmarska /4804	Komisja Bioetyczna Pomorska Akademia Medyczna 70-204 Szczecin	Prof. Tadeusz Brzeziński	26 April 04	25 April 05	submitted on the 19 Oct 05 with stamp of 25 Oct 05 (silent approval)
Dr Andrzej Horban /4805	Komisja Bioetyczna Centrum Medycznego Kształcenia Podyplomowego 01-813 Warsaw	Prof. Ewa Marcinowska-Suchowierska	03 March 04	11 May 05	submittend on the 19 Oct 05 with stamp of 20 Oct 05 (silent approval)

ROMANIA IRBs / Investigator					
**Investigator/ Study Site**	**IRB or Ethics Committee**	**IRB or EC Chairman**	**Date of Final Approval**		
			**Protocol**	**Amendment 1**	**Amendment 2**
for both sites	National Ethics Commission	George Litarczek	30 January 04	05. April 05	01 November 05
	National Medicines Agency (Authority)	Magdalena Badulescu	13February 04	31. March .05	21 November 05
Dan Duiculescu/4012	Local Ethics Committee "Dr.Victor Babes" Hospital of Infectious and Tropical Diseases	Emanoil Ceausu	26 January 05 notification	05 May 05 notification	04 October 05 notification
Adrian Streinu Cercel/4013	Local Ethics Committee "Prof.Dr.Matei Bals" Institute of Infectious Diseases	Monica Luminos	23 January 05 notification	18 April 05 notification	27 September 05 notification

Russia IRBs / Investigator					
**Investigator/ Study Site**	**IRB or Ethics Committee**	**IRB or EC Chairman**	**Date of Final Approval**		
			**Protocol**	**Amendment 1**	**Amendment 2**
Prof.Vinogradova / 0701	St.Petersburg City AIDS and Infectious Diseases Prevention, Ethical Committee	Raisa Shapovalenko	NA	NA	NA
Prof. Pokrovsky / 0702	Central Scientific Research Institute of Epidemiology, Ethical Committee	Vadim Mashilov	NA	NA	NA
Russia	Ethical Committee at the Federal Department of Drug Quality, Efficacy and Safety Control	Fedor Komarov	25 February 04	01 March 05	05 Oct 05

SPAIN IRBs / Investigator					
**Investigator/ Study Site**	**IRB or Ethics Committee**	**IRB or EC Chairman**	**Date of Final Approval**		
			**Protocol**	**Amendment 1**	**Amendment 2**
Dr. Gatell / 3401	Comité de Investigación Clínica del Hospital Clinic i Provincial de Barcelona	D. Miguel Angel Asenjo Sebastián	02 March 04	12 May 05	30 September 05
Dr. Clotet / 3402	Comité de Investigación Clínica del Hospital Universitario Germans Trias i Pujol	Dª. M. Villagrasa Serrano	12 March 04	11 March 05	30 September 05
Dr. Podzamdcer / 3403	Comité de Investigación Clínica de la Ciudad Sanitaria y Universitaria de Bellvitge	Dª. Ignacia Ferrer	05 April 04	31 March 05	27 October 05

SPAIN IRBs / Investigator					
**Investigator/ Study Site**	**IRB or Ethics Committee**	**IRB or EC Chairman**	**Date of Final Approval**		
			**Protocol**	**Amendment 1**	**Amendment 2**
Dr. Gurguí / 3404	Comité de Investigación Clínica del Hospital de la Santa Creu i Sant Pau	D. Jose Luis Barrio Medrano	25 February 04	23 February 05	30 September 05
Dr. Dalmau / 3405	Comité de Investigación Clínica del Hospital Mutua Terrassa	D. Ramón Pla	08 March 04	24 February 05	04 October 05
Dr. Rubio and Dr. Pulido / 3406	Comité de Investigación Clínica del Hospital 12 de Octubre	Dª. María Ugalde Diez	16 March 04	29 March 05	05 October 05
Dr. Gonzalez / 3407	Comité de Investigación Clínica del Hospital Carlos III	D. Carlos Lahoz Rallo	02 March 04	N.A.	N.A.

SPAIN IRBs / Investigator					
**Investigator/ Study Site**	**IRB or Ethics Committee**	**IRB or EC Chairman**	**Date of Final Approval**		
			**Protocol**	**Amendment 1**	**Amendment 2**
Dr. Moreno / 3408	Comité de Investigación Clínica del Hospital Ramón y Cajal	D. Javier Zamora Romero	09 March 04	N.A.	N.A.
Dr. Viciana / 3409	Comité Regional de ensayos clínicos	D. Eduardo de Teresa Galván	04 February 04	31 March 05	30 September 05
Dr. Márquez / 3411	Comité Regional de ensayos clínicos	D. Eduardo de Teresa Galván	04 February 04	31 March 05	30 September 05
Dr. Gómez-Sirvent /3412	Comité de Investigación Clínica del Hospital Universitario de Canarias	Dª. Julia Nazco Casariego	18 March 04	07 April 05	30 September 05

Thailand IRBs / Investigator					
**Investigator/ Study Site**	**IRB or Ethics Committee**	**IRB or EC Chairman**	**Date of Final Approval**		
			**Protocol**	**Amendment 1**	**Amendment 2**
all sites	The Ethical Review Comité for Research in Human subjects Ministry of Public Health	Seree Tuchinda	11 June 04	06 May 05	09 November 05
Dr. Kiat Ruxrungtham / 6601	Chulalongkorn University	Prof. Anek Aribarg	06 September 04	27 April 05	10 Oct 05
Dr. Sasisopin Kiertiburanakul / 6603	Mahidol University Bangkok	Krisada Ratana-olarn	19 May 04	29 September 05	19 Oct 05

UK IRBs / Investigator					
**Investigator/ Study Site**	**IRB or Ethics Committee**	**IRB or EC Chairman**	**Date of Final Approval**		
			**Protocol**	**Amendment 1**	**Amendment 2**
Dr Jonathan Ainsworth/ 4422	Barnet, Enfield and Haringey LREC	Dr Peter Savage	10 May 04	Approved by MREC 03 March 05. LREC approval not required	Approved by MREC 02 Nov 05. LREC approval not required
Dr Margaret Johnson / 4409	Royal Free and Medical School LREC	Dr Michael Pegg	10 May 04	Approved by MREC 03 March 05. LREC approval not required	Approved by MREC 02 Nov 05. LREC approval not required
Dr Clifford Leen / 4412	Lothian LREC	Mr N Grier or Prof P Hayes or Dr C West or Dr M Logan	10 May 04	Approved by MREC 03 March 05. LREC approval not required	Approved by MREC 02 Nov 05. LREC approval not required
